# Molecular mechanism and therapeutic potential of HDAC9 in intervertebral disc degeneration

**DOI:** 10.1186/s11658-023-00517-x

**Published:** 2023-12-13

**Authors:** Ming Lei, Hui Lin, Deyao Shi, Pan Hong, Hui Song, Bomansaan Herman, Zhiwei Liao, Cao Yang

**Affiliations:** Department of Orthopedics, Union Hospital, Tongji Medical College, Huazhong University of Science and Technology, 1277 Jiefang Avenue, Wuhan, 430022 China

**Keywords:** Intervertebral disc degeneration, HDAC9, RUNX3, Cell viability, Apoptosis

## Abstract

**Background:**

Intervertebral disc degeneration (IVDD) is the major cause of low-back pain. Histone deacetylase 9 (HDAC9) was dramatically decreased in the degenerative nucleus pulposus (NP) samples of patients with intervertebral disc degeneration (IVDD) according to bioinformatics analysis of Gene Expression Omnibus (GEO) GSE56081 dataset. This study aims to investigate the role of HDAC9 in IVDD progression.

**Methods:**

The contribution of HDAC9 to the progression of IVDD was assessed using HDAC9 knockout (HDAC9^KO^) mice and NP-targeted HDAC9-overexpressing mice by IVD injection of adenovirus-mediated HDAC9 under a Col2a1 promoter. Magnetic resonance imaging (MRI) and histological analysis were used to examine the degeneration of IVD. NP cells were isolated from mice to investigate the effects of HDAC9 on apoptosis and viability. mRNA-seq and coimmunoprecipitation/mass spectrometry (co-IP/MS) analysis were used to analyze the HDAC9-regulated factors in the primary cultured NP cells.

**Results:**

HDAC9 was statistically decreased in the NP tissues in aged mice. HDAC9^KO^ mice spontaneously developed age-related IVDD compared with wild-type (HDAC9^WT^) mice. In addition, overexpression of HDAC9 in NP cells alleviated IVDD symptoms in a surgically-induced IVDD mouse model. In an in vitro assay, knockdown of HDAC9 inhibited cell viability and promoted cell apoptosis of NP cells, and HDAC9 overexpression had the opposite effects in NP cells isolated from HDAC9^KO^ mice. Results of mRNA-seq and co-IP/MS analysis revealed the possible proteins and signaling pathways regulated by HDAC9 in NP cells. RUNX family transcription factor 3 (RUNX3) was screened out for further study, and RUNX3 was found to be deacetylated and stabilized by HDAC9. Knockdown of RUNX3 restored the effects of HDAC9 silencing on NP cells by inhibiting apoptosis and increasing viability.

**Conclusion:**

Our results suggest that HDAC9 plays an important role in the development and progression of IVDD. It might be required to protect NP cells against the loss of cell viability and apoptosis by inhibiting RUNX3 acetylation and expression during IVDD. Together, our findings suggest that HDAC9 may be a potential therapeutic target in IVDD.

**Graphical Abstract:**

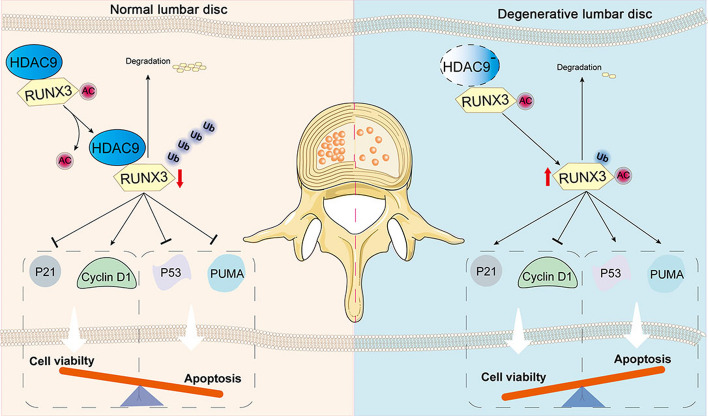

**Supplementary Information:**

The online version contains supplementary material available at 10.1186/s11658-023-00517-x.

## Introduction

Intervertebral disc degeneration (IVDD) is the major cause of low-back pain (LBP) in the elderly [1, 2]. LBP causes physical disability and psychological health impairment, which leads to poor quality of life and economic burden. Given the continuous increase in the population of individuals aged over 60 years, LBP may result in a great burden on global health [3]. Therefore, it is urgent and necessary to understand the pathogenesis of IVDD and find new therapeutic targets to ameliorate the progression of IVDD.

The intervertebral disc (IVD) is composed of three tissue compartments: the central nucleus pulposus (NP), the circumferential annulus fibrosus (AF), and the cartilaginous endplates (EP). NP is a hydrogel-like tissue and is rich in type II collagen (collagen II, Col2a1) and proteoglycans. The main role of NP is to confer the resistance to compressive loads [4–6]. AF is primarily composed of type I collagen and proteoglycan aggregates and is responsible for resisting the swelling force from NP by surrounding it [4, 7]. EP situates at the articular surface of the disc and the adjacent vertebrae to cap the disc and provide the water and nutrients with the support of the adjacent vertebral capillary bed [8, 9]. These compartments together provide spinal flexibility and stability and transmit compressive loads [10]. Therefore, it is very important to maintain the stable organizational structure of IVD. Disc degeneration is an inevitable consequence of aging. The structural failure of the IVD such as the appearance of clefts and tears in NP and EP, loss of annulus-nucleus boundary, and AF extension into NP frequently occurs in aging/degenerating discs [4]. Recent studies have shown that the development of intervertebral disc degeneration (IVDD) is primarily caused by disc cell death, excessive secretion of inflammatory cytokines, and degradation of extracellular matrix components such as proteoglycan and collagen II [11–17].

Protein posttranslational modifications such as acetylation and ubiquitination have important roles in gene expression. Particularly, histone acetylation and non-histone protein acetylation mainly occur at lysine residues and are involved in regulating gene expression and biological function [18–20]. The enzyme histone acetyltransferases (HATs) and histone deacetylases (HDACs) respectively regulate the acetylation and deacetylation of non-histone proteins including transcription factors to control the protein stability and transcriptional activity [19–21]. HDACs are a family of enzymes that remove the acetyl from the histones or non-histone proteins. They can be grouped into three classes based on the structure and homology: class I (HDAC1–3 and HDAC8), class II (HDAC4–10) and class III (SIRT1–7) [22]. HDAC9 belongs to class II and has a variety of physiological functions [19, 23–26]. Interestingly, HDAC9 was found to be differently expressed in NP tissues of patients with degenerated IVD according to bioinformatics analysis of IVDD genome microarray based on Gene Expression Omnibus (GEO) dataset GSE56081. Prior literature suggests that HDAC9 can promote cell proliferation and inhibit cell apoptosis in vitro [25, 27–29]. Therefore, we proposed that HDAC9 might play a role in IVDD development by regulating cell viability and apoptosis.

In the current study, we found that HDAC9 was downregulated in IVD tissues of degenerative IVD patients through analysis of public datasets. Cell death is exacerbated in IVD tissues of 6-month-old HDAC9 knockout (HDAC9^KO^) mice. Overexpression of HDAC9 under a Col2a1 promoter attenuates intervertebral disc damage in a surgically induced IVDD model. Mechanistic studies revealed that HDAC9 physically interacts with runt-related transcription factor 3 (RUNX3) to repress RUNX3 acetylation and expression, thereby preventing apoptosis and increasing cell viability in NP cells.

## Materials and methods

### HDAC9 knockout mice

The animals were kept at 22 ± 1 °C in a specific pathogen-free facility and on a 12 h light/dark cycle with free access to food and water. C57BL/6J mice (product number, 11001A) were purchased from Beijing HFK Bioscience (Beijing, China). HDAC9 knockout mice (HDAC9^KO^) were constructed by Wanleibio (Shenyang, China). Briefly, the HDAC9^KO^ mice were generated by the CRISPR/cas9 system and two small guide RNAs (sgRNA-1 and sgRNA-2) targeting the *HDAC9* gene deletion of exons 3–6 in mice of C57BL/6J background. The targeting nucleotide sequences for constructing sgRNAs were as follows:

sgRNA-1, 5′-GTGCTTACATGCGCTATTCA-3′,

sgRNA-2, 5′-AAGCTCGGGCATTCTGCCCT -3′.

Female C57BL/6J mice (6 weeks old) were superovulated with 5 IU pregnant mare serum gonadotrophin (PMSG) and 5 IU human chorionic gonadotropin (HCG) [intraperitoneal (i.p.)]. The eggs from oviducts of female C57BL/6J mice and sperms from male C57BL/6J mice (12 weeks old) were collected for fertilization in vitro. Subsequently, the zygotes were microinjected with the prepared two sgRNAs targeting gene deletion of exons 3–6 of mouse *HDAC9* and Cas9 protein and surgically transferred into the oviducts of pseudo-pregnant female mice. The offspring (3 weeks old) generation mutant was identified from tail DNA samples by genotyping polymerase chain reaction (PCR) and validated by sequencing analysis. Heterozygous HDAC9^+/−^ mice (F0) were bred with wild-type (WT) mice and then HDAC9^+/−^ female mice (F1) were further bred with HDAC9^+/−^ male mice (F1) to generate HDAC9^−/−^ (HDAC9^KO^) mice (F2). *HDAC9* gene deletion in HDAC9^KO^ mice was validated using tail DNA samples by sequencing analysis.

### Number of experimental animals

A total of 108 mice, including wild-type C57BL/6J mice, HDAC9^KO^ mice and age-matched wild-type littermate control (HDAC9^WT^) mice, were used in an age-related IVDD model and a surgically-induced IVDD model. The number of experimental mice in each experiment was described in the following sections.

### Age-related IVDD model

To understand the role of HDAC9 in IVDD in the aging-related pattern, a total of 12 wild-type C57BL/6J mice were randomly divided into two groups (6 mice per group), and the magnetic resonance imaging (MRI) examination, histological and molecular changes in IVDs were assessed at 6 and 18 months. To reveal the consequences of the reduced expression of HDAC9 in degenerated IVD, HDAC9^KO^ mice and HDAC9^WT^ mice (a total of 48 mice, 24 mice per group: 6 mice per group for MRI and histopathologic examination at each timepoint and 6 mice per group for immunoprecipitation assay at 6 months of age), at 1, 3, and 6 months of age, were weighed and underwent MRI examination using a 3.0 T MRI system (GE Discovery, MR750). Subsequently, the spinal segments of mice were removed for the histopathologic examination and molecular biology experiments.

### Surgically induced IVDD model

To investigate the role of HDAC9 in NP cells, adenovirus overexpression vector pDC315 (VT1500, Youbio, Chongqing, China) was inserted with Col2a1 promoter used to construct adenovirus-mediated overexpression of HDAC9 (HDAC9^Col2a1^) and negative control adenovirus (NC^Col2a1^). Surgically induced IVDD model was performed as previously reported [30]. Briefly, 8-week-old male C57BL/6J mice were divided into four groups including sham, IVDD, IVDD + NC^Col2a1^, and IVDD + HDAC9^Col2a1^ (a total of 48 mice, 12 mice per group: 6 mice per group for MRI and histopathologic examination and 6 mice per group for immunoprecipitation assay). After anesthesia, the spine was made visible using an anterior midline transperitoneal approach. The L4, L5, and L6 vertebral bodies were exposed after separating the psoas major muscles and hind peritoneum. IVDD mice were punctured at the L5/6 IVD with a 30-gauge needle. Mice from IVDD + NC^Col2a1^ and IVDD + HDAC9^Col2a1^ groups were injected with 5 µl of NC^Col2a1^ or HDAC9^Col2a1^ (2 × 10^11^ pfu/ml) during needle puncture. The mice from the sham group were subjected to the same procedures as that in the IVDD group only without needle puncture. After the procedure, the incision was stitched up, and the animals were allowed to move freely in cages. After 4 weeks of surgery, MRI detection was performed and L5/6 IVD tissue was collected for testing.

### NP cell isolation and culture

The NP cells from the lumbar discs were isolated from male wild-type C57BL/6J mice, HDAC9^KO^ mice, and matched-HDAC9^WT^ mice (8 weeks old). In brief, mice were euthanized and the entire spine was separated. Subsequently, the lumbar IVDs were exposed and the white and gelatinous NP tissue was carefully separated from AF tissue. The NP tissue was washed with phosphate-buffered saline (PBS) several times and cut into pieces, and treated with 0.2% type II collagenase (Biosharp, China, BS164) for 4 h. After filtering through the 100-μm mesh, the cell suspension was centrifuged at 350 g for 10 min. Then the cells were cultured in Dulbecco’s modified eagle medium (DMEM) (Servicebio, Wuhan, China, G4510) supplemented with 10% fetal bovine serum (FBS; Tianhang, China, 11,011–8611) and incubated in a humidified atmosphere (5% CO_2_, 37 °C). When the NP cells were 80% confluent, the cells were subcultured and identified using anti-collagen II antibody by immunofluorescence assay.

### Cell infection and treatment

For the purpose of HDAC9 knockdown/overexpression and RUNX3 knockdown in NP cells, the lentivirus containing short hairpin RNA of HDAC9 (Lt.shHDAC9), RUNX3 (Lt.shRUNX3) and negative control (Lt.shNC), adenovirus-mediated overexpression of HDAC9 (Ad.HDAC9) and control (Ad.NC) were constructed and packed. NP cells were infected with Lt.shNC, Lt.shHDAC9, or co-infected with Lt.shHDAC9 + Lt.shNC or Lt.shHDAC9 + Lt.shRUNX3 for 72 h and subjected to further experiments. To determine whether HDAC9 regulates RUNX3 protein stability, Lt.shHDAC9-infected cells were treated with protein synthesis inhibitor cycloheximide (CHX, 20 µg/ml, MCE, USA, HY-12320) and the protein expression of RUNX3 was measured at 0, 4, 8, and 12 h post-CHX treatment. In addition, we tested whether the degradation of RUNX3 was via the ubiquitin–proteasome system. After Lt.shHDAC9 infection, a proteasome inhibitor MG132 (10 µM, Aladdin, M126521) was added into each well and cultured for 8 h, after which the cells were subjected to co-IP and western blot assay. To confirm HDAC9 regulating viability and apoptosis of NP cells, HDAC9^KO^ NP cells were infected with Ad.HDAC9 or Ad.NC. After infection for 72 h, the cells were subjected to further experiments.

### Data source and analysis

The GSE56081 dataset, including mRNA profiling analysis of five human control NPs and five degenerative NP tissues, was downloaded from the GEO database (https://www.ncbi.nlm.nih.gov/geo/)s. The differentially expressed genes (DEGs) were analyzed using GEO2R online analytical tool (http://www.ncbi.nlm.nih.gov/geo/geo2r) and obtained from the additional files published by Lan et al. [31]. Genes that met the thresholds of |log_2_FC| ≥ 1 and *p* value < 0.05 were considered DEGs. Enrichment analysis to determine the gene functions and biological pathways of all the genes from the GSE56081 dataset was performed by gene set enrichment analysis (GESA) based on the Gene Ontology (GO) analysis and Kyoto Encyclopedia of Genes and Genomes (KEGG) analysis using the “R package.” Gene functions are classified into three GO terms including biological process (BP), cellular component (CC), and molecular function (MF). The genes related to protein modification such as ubiquitination, deubiquitination, phosphorylation, dephosphorylation, acylation, and deacylation were downloaded from The Gene Ontology resource (GOR) knowledge base (http://geneontology.org). The common genes from the GOR knowledge base and the DEGs were screened and displayed using a raincloud plot. Heatmap was used to present the top three upregulated and downregulated DEG functions as ubiquitination, deubiquitination, phosphorylation, dephosphorylation, acylation, and deacylation. UpSet plot (|cor|> 0.8, and *p* value < 0.05) presented the intersection of these six deacetylation genes and the DEGs from the GSE56081 dataset.

### mRNA-Seq analysis

The total RNA of NP cells isolated from the HDAC9^KO^ and HDAC9^WT^ mice (*n =* 3) was extracted using TRIpure reagent (BioTeke, China, RP1001). The mRNA sequencing (mRNA-seq) was performed and analyzed by Novogene Co., Ltd (Beijing, China). The raw data were recorded in the FASTQ, and clean data (clean reads) were obtained by removing the reads with adapter contamination, low-quality, and high uncertain bases (˃10%) from raw data. DEG analysis was performed using the R package. Genes that met the thresholds of |log_2_FC| ≥ 1.0 and *p* value < 0.05 were considered as DEGs. The volcano plot and heatmap were drawn, and GSEA enrichment analysis was performed. Additionally, GSEA analysis based on GO terms including oxidative phosphorylation, deubiquitinase activity, histone acetyltransferase activity, deacetylase activity, and KEGG pathways, including cytokine–cytokine receptor interaction, PI3K-Akt signaling pathway, and glycolysis/gluconeogenesis, were analyzed.

### Coimmunoprecipitation/mass spectrometry (co-IP/MS)

To screen out the interacting proteins of HDAC9, we conducted co-IP/MS and analyzed the HDAC9 antibody precipitated proteins in NP cells. Briefly, NP cells were lysed in western and IP lysis buffer (Beyotime, Shanghai, China, P0013). Protein A + G agarose gel (Beyotime, P2197) was incubated with anti-HDAC9 antibody (Santa Cruz Biotechnology, CA, USA, Sc-398003) or anti-IgG (Beyotime, A0208) for 30 min. After washing and centrifuging, antibody-enriched protein A + G agarose gel was incubated with supernatant of the cell lysate for 2 h. Subsequently, the co-IP products were separated in sodium dodecyl sulfate (SDS)–polyacrylamide gel and analyzed using liquid chromatography (LC)–MS with the assistance of Qinglianbio (Beijing, China). LC–MS detection was undertaken using an L-3000 HPLC system (RIGOL, Beijing, China) and Orbitrap Eclipse mass spectrometer (Thermo Fisher Scientific). Database screening was performed against the Mus musculus database using Proteome Discoverer 2.4 (Thermo Fisher Scientific). The precursor mass tolerance is ± 15 ppm, and the fragment ion mass tolerance is ± 0.02 Da. Trypsin was specified as the digestive enzyme, and two missed cleavages were allowed. A total of 2356 proteins interacted with HDAC9 and 581 proteins interacted with IgG. The datasets of proteins containing acetylation sites, phosphorylation sites, and ubiquitination sites were downloaded from https://www.phosphosite.org/homeAction.action (PhosphoSitePlus database). We identified the HDAC9-interacting proteins that can be possibly modified by acetylation, phosphorylation, and ubiquitination. Moreover, a total 1907 specific HDAC9-interacting proteins in NP cells were functionally classified according to GO and eukaryotic orthologous groups (KOGs) of protein annotations.

In other assays, IVD tissues or NP cells were lysed and incubated with anti-RUNX3 antibody (CST, MA, USA, 13089) or anti-IgG (Beyotime, A0208), followed by IP assay. Subsequently, the precipitated proteins were incubated with an anti-HDAC9 antibody (1:5000, Abcam, Cambridge, UK, ab109446), anti-RUNX3 antibody, anti-acetyl lysine antibody (1:1000, Abcam, ab190479), or anti-ubiquitin (Ubi) antibody (1:5000, Abcam, ab134953).

### Histological analysis

The IVD degeneration was assessed by Safranin-O/Fast green staining. Briefly, lumbar spines collected from mice were fixed with 4% paraformaldehyde (Sinopharm Chemical Reagent, China, 80,096,618), followed by paraffin embedding. Subsequently, 5-μm mid-coronal sections of IVDs were prepared. The sections were then stained with 0.2% safranin-O/2% fast green (Servicebio, China, G1053-100ML). The stained sections were visualized by a microscope (Olympus, BX53, Japan) and pictured using the imaging system (Olympus, DP73) and dedicated software. The degeneration of the sections was histologically scored [32]. The areas of NP cell band, NP, and IVD were measured by tracing in the imaging system, and the area ratios of NP cell band/IVD and NP/IVD were calculated.

## Immunofluorescence assay

The expression of HDAC9, cyclin D1, p21, p53, and RUNX3 in NP tissue and NP cell identification using collagen II were determined by immunofluorescence. Briefly, coronal 5-μm sections and cell slides were blocked by 1% bovine serum albumin (BSA; Sangon Biotech, China, A602440-0050) or goat serum (Solarbio, Beijing, China, SL038). The sections and cells were washed with PBS and incubated with the primary antibody. The primary antibodies used were anti-HDAC9 (1:100, ab109446), anti-RUNX3 (1:50, Abcam, ab135248), anti-p21 (1:100, Proteintech, Wuhan, China, 10,355–1-AP), anti-p53 (1:100, Proteintech, 60,283–2-Ig), anti-cyclin D1 (1:100, Proteintech, 26,939–1-AP), and anti-collagen II (1:100, Affinity, AF0135) at 4 °C overnight. Tissue sections and cells were washed with PBS and incubated with the appropriate second antibodies for 1 h at room temperature. For double staining of HDAC9 and RUNX3 in cultured NP cells, the NP cells were incubated with anti-HDAC9 (1:50, ab109446) and anti-RUNX3 (1:50, ab135248), and subsequently stained with the appropriate second antibodies. After washing with PBS, the sections and cell slides were counterstained with DAPI (Aladdin, China, D106471-5 mg), and the percentages of positive staining cells were quantified using a fluorescence microscope with image software (Olympus).

### Terminal deoxynucleotidyl transferase dUTP nick-end labeling (TUNEL) assay

The cell death in IVD tissues was measured using an “in situ cell death detection” Kit (Roche, 12,156,792,910). Sections were permeabilized with 0.1% Triton X–100 for 8 min at room temperature and stained with TUNEL solution (Enzyme solution: Label Solution of 1:9) for 60 min. Subsequently, sections were subjected to DAPI staining and visualization using a fluorescence microscope.

### CCK-8 cell viability assay

Cell viability of NP cells was evaluated with a CCK-8 assay. In brief, NP cells (4000/well) were added to a 96-well plate. After 72 h infection, 10 μl of CCK-8 reagent (Solarbio, CA1210) was added into each well. The absorbance of each well at 450 nm was measured using a microplate reader (Biotek, Vermont, USA, 800TS) after 2 h incubation.

### Quantitative real-time reverse transcription polymerase chain reaction (RT–qPCR)

The lumbar IVD tissues collected from mice and isolated NP cells were used to extract total RNA using TRIpure reagent. The concentration of total RNA was measured and equal amounts of RNA were subjected to reverse transcriptase to synthesize cDNA using a reverse transcriptase system containing BeyoRT II M-MLV (Beyotime, D7160L). RT–qPCR was performed using SYBR Green (Solarbio, SY1020) and 2 × Taq PCR MasterMix (Solarbio, PC1150) according to the manufacturer’s instructions. The forward and reverse primer sequences were as follows:

*HDAC9*, Forward 5′-CAGAATCCTCGGTCAGTAG-3′,

Reverse 5′-GTTAGAAGCATTGAGTGGG-3′;

*RUNX3*, Forward 5′-CAACGCTTCCGCTGTCA-3′,

Reverse 5′-GCCTTGGTCTGGTCTTCTATC-3′;

*p21*, Forward 5′-ACTTCCTCTGCCCTGCTGC-3′,

Reverse 5′-GCTGGTCTGCCTCCGTTTT-3′;

*CyclinD1*, Forward 5′-TGAGGAGCAGAAGTGCGAAGA-3′,

Reverse 5′-CGGCAGTCAAGGGAATGGT-3′;

*PUMA*, Forward 5′-TGTCACCAGCCCAGCAGCAC-3′,

Reverse 5′-GTTGAGGTCGTCCGCCATCC-3′.

The results were calculated using the 2^−ΔΔCt^ method and the relative expression of each gene to β-actin was calculated.

### Apoptosis assay

The apoptosis of NP cells was measured using an annexin V-FITC/propidium iodide (PI) staining assay (Solarbio, CA1020). In brief, after infection, cells were collected and washed with PBS. The resuspended cells were incubated with annexin V-FITC (10 μl) and propidium iodide (10 μl) in a binding buffer in the dark for 15 min. The ratio of apoptotic cells was evaluated by gating annexin V-positive cells by flow cytometry (Aceabio, Novocyte, USA).

### Western blot analysis

The IVD tissues and NP cells were digested using western and IP lysis buffer (Beyotime, P0013) supplemented with phenylmethylsulfonyl fluoride (PMSF) (Beyotime, ST506). Subsequently, the lysates were centrifugated at 10000*g* for 5 min at 4 °C, and the supernatant was retained. Following quantification using a BSA protein assay kit (P0011, Beyotime), equal amounts of proteins (20–40 mg) were separated by SDS–polyacrylamide gels and transferred onto a polyvinylidene difluoride (PVDF) membrane (Millipore, IPVH00010). Then the proteins were blotted with anti-HDAC9 (1:5000, ab109446), anti-RUNX3 (1:1000, CST, #13,089), anti-p21 (1:500, Abclonal, China, A2691), anti-p53 (1:1000, Abclonal, A11232), anti-PUMA (1:1000, Abcam, ab9643), and anti-cyclin D1 (1:1000, Abclonal, A19038) overnight at 4 °C. The β-actin was used as an internal reference. After being incubated with second antibodies, the blots were visualized using a protein electrophoresis apparatus (BEIJINGLIUYI, China, DYCZ-24DN) and grey value analysis was conducted using Gel-Pro Analyzer.

### Statistical analyses

GraphPad Prism 8 (GraphPad Software Inc., USA) was used for statistical analyses. Differences between two groups were analyzed using the Student’s *t*-test and differences among more than two groups were analyzed using the one-way ANOVA with a Tukey’s multiple comparison test. Data are presented as the mean ± standard deviation (SD). A value of *p* < 0.05 was considered to be significant.

## Results

### Protein modifications play an important role in the progression of IVDD

**Fig. 1:**
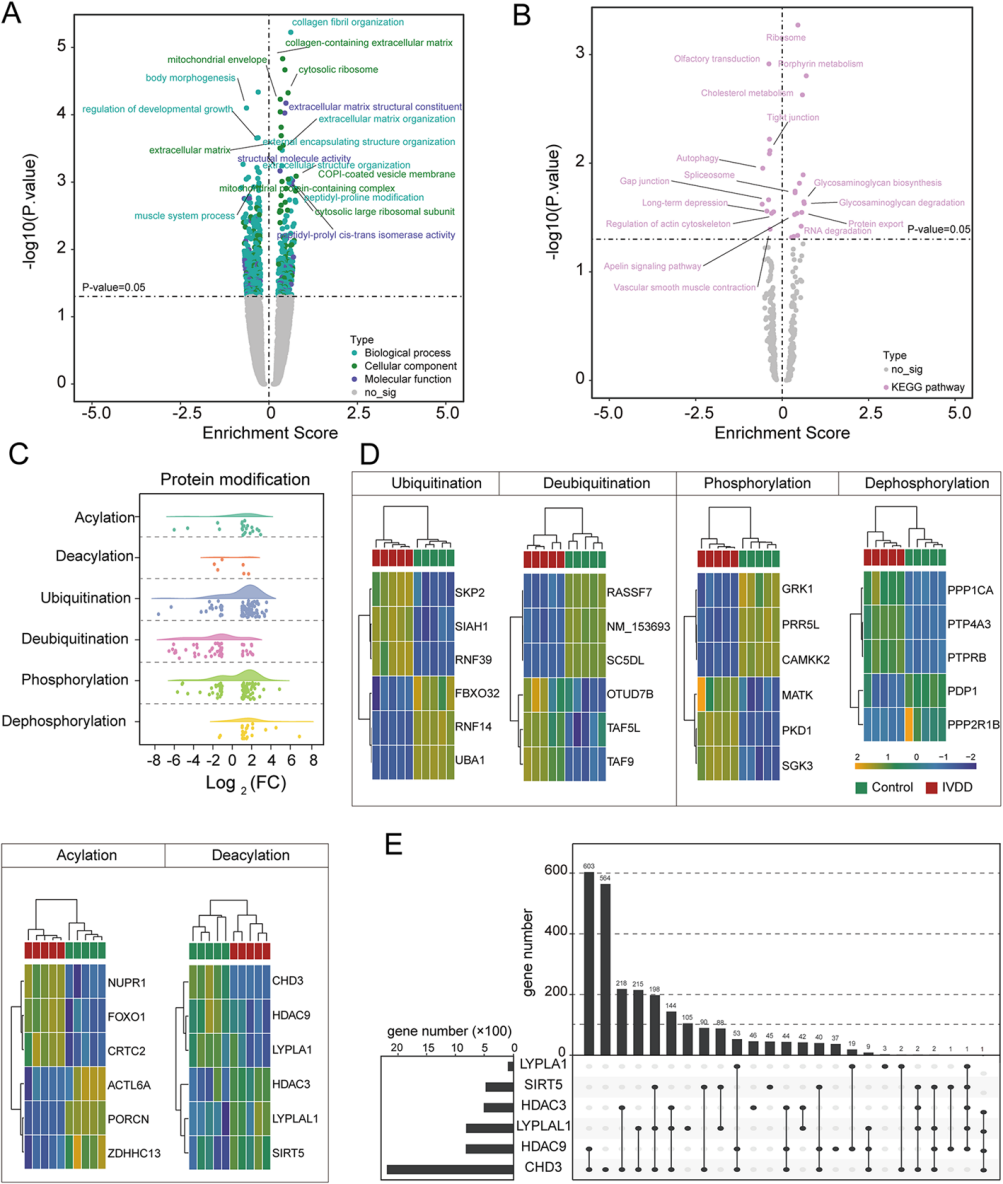
Decreased expression of HDAC9 in degenerated IVD in patients. **A**, **B** GSE56081 dataset (NP tissues from patients with IVDD and normal controls [31]) were analyzed by gene set enrichment analysis (GSEA) based on the Gene Ontology (GO) analysis and Kyoto Encyclopedia of Genes and Genomes (KEGG) analysis. **C** Raincloud plot exhibited DEGs related to protein modification such as ubiquitination, deubiquitination, phosphorylation, dephosphorylation, acylation, and diacylation based on the Gene Ontology resource knowledge base (Raincloud plot was drawn using https://www.chiplot.online/). **D** Heatmap presented the top three upregulated and downregulated DEGs from **C**. **E** UpSet plot displayed the intersections between six deacetylation genes and DEGs from the GSE56081 dataset

We analyzed the data of control and degenerative NP samples from the GSE56081 dataset and published data [31]. The gene functions (GO) and related biological pathways (KEGG enrichment) of genes using GSEA were evaluated and several top significantly enriched GO terms and KEGG pathways were exhibited in Fig. 1A and B. We wondered whether the DEGs participate in protein modification. The results showed that several genes related to ubiquitination, phosphorylation, and acylation were significantly changed in degenerative NP tissues compared with controls (Fig. 1C). Heatmap presented the top three upregulated and downregulated DEGs in each protein modification, and the expressions of *CHD3*, *HDAC9*, *LYPLA1*, *HDAC3*, *LYPLAL1*, and *SIRT5* were significantly changed in NP cells. These genes are related to deacylation (Fig. 1D). HDAC9 attracted our attention because it was the most significantly differentially expressed in these six genes, and upset plot showed that about 800 DEGs were related with HDAC9 in NP cells (Fig. 1E, Additional file 1: Table S1). Therefore, we focused on the roles of HDAC9 in this study.

### HDAC9 is decreased in degenerated IVD in aged mice

**Fig. 2:**
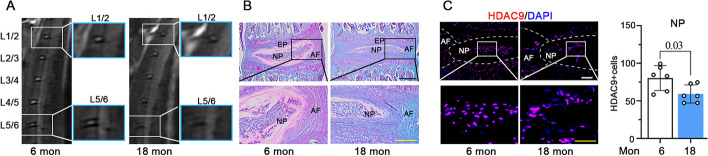
HDAC9 is decreased in degenerated IVD in aged mice. **A** MRI scan of the lumbar spine and intervertebral discs (IVDs) of 6-month and 18-month-old mice. **B** Coronal sections of disc compartments stained by safranin-O/fast green. Scale bar: black, 500 μm; yellow, 200 μm. **C** Fluorescence staining of HDAC9 in disc compartments. Scale bar: White, 100 μm; Yellow, 50 μm. Quantification of staining for HDAC9-positive cells. Data are represented as mean ± SD (*n* = 6). A *p*-value of less than 0.05 was considered significant using an unpaired student’s *t*-test. NS: not significant

We detected the expression of HDAC9 in IVD tissues from C57BL/6J mice at 6 and 18 months. Compared with 6-month-old mice, an MRI scan showed that the degradation occurred at lumbar IVDs (L1/2, L2/3, L3/4, L4/5, L5/6) in 18-month-old mice. The structure of the IVDs was inhomogeneous with a lower intense white signal (L1/2, L2/3, and L3/L4) or an intermediate gray signal intensity (L4/5 and L5/6) in aged mice. Moreover, the boundary between NP and AF in L4/5 and L5/6 was unclear (Fig. 2A). Notable degenerative changes in IVDs, including proteoglycan loss, increased matrix fibrosis, and NP cell band disruption, were seen in 18-month-old mice by safranin-O/fast green staining (Fig. 2B). Immunofluorescence staining showed that HDAC9 was widely distributed in IVD tissues of 6-month-old mice. However, HDAC9-positive cells were dramatically reduced in IVD tissues of 18-month-old mice, especially in NP (Fig. 2C). The results indicated that HDAC9 might play an important role in the procession of IVDD and affect the function of NP cells.

### Deficiency of HDAC9 contributes to the degeneration of IVD during aging

**Fig. 3:**
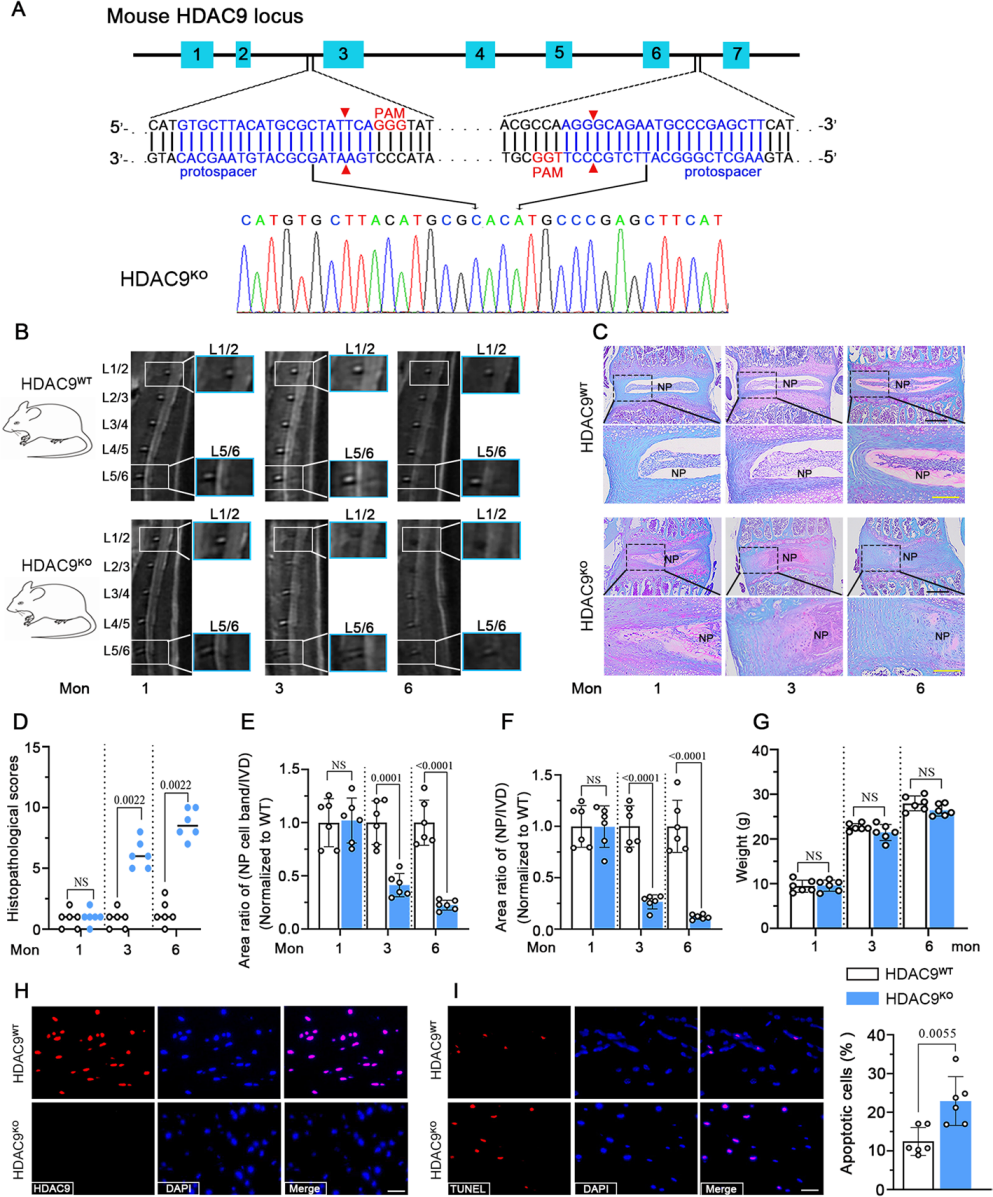
Deletion of HDAC9 contributes to degeneration of IVD during aging. **A** Schematic diagram of the HDAC9 knockout design showing partial mouse *HDAC9* gene (top), sequences of two gRNAs used for deletion of exon 3 to 6 (middle), and the truncated DNA was confirmed by sequencing validation (bottom). **B** MRI scan of the lumbar spine and IVDs of HDAC9^WT^ mice and HDAC9^KO^ mice at 1, 3, and 6 months. **C**, **D** Coronal sections of disc compartments stained and analyzed by safranin-O/fast green. Scale bar: black, 500 μm; yellow, 200 μm. Quantification of area ratios of NP cell band/IVD (**E**) and NP/IVD (**F**). **G** Body weight of WT mice and HDAC9^KO^ mice at 1, 3, and 6 months. **H** Fluorescence staining of HDAC9 in NP compartment. Scale bar: 25 μm. **I** Apoptotic cells in the NP compartment were detected by TUNEL staining. Scale bar: 25 μm. Data are represented as mean ± SD (*n* = 6). A *p* value of less than 0.05 was considered significant using an unpaired Student’s *t*-test. NS, not significant

HDAC9^KO^ mice were used to reveal the role of HDAC9 in IVDs. Two sgRNAs were designed to target intron 2 and intron 6 of the mouse *HDAC9* gene and the truncated DNA deleting exon 3 to exon 6 from the HDAC9^KO^ mice was confirmed by sequencing validation (Fig. 3A). The IVDs of HDAC9^KO^ mice and HDAC9^WT^ mice were assessed by MIR scan. The inhomogeneous structure of L1/2 and L5/6 with intermediate gray signal intensity occurred in HDAC9^KO^ mice from 3 months of age. Moreover, HDAC9^KO^ mice exhibited more severe degenerative changes of IVDs in the structure, signal intensity, and boundary between NP and AF than WT mice at 6 months of age (Fig. 3B). The safranin-O/fast green staining confirmed that the degeneration of IVDs in HDAC9^KO^ mice with aging. With age increasing, the matrix of the inner AF became degenerated with loosely packed collagen fibers and the matrix of NP changed from a gel-like structure to a fibrotic one accompanied by the loss of NP. The increase of proteoglycan content in inner AF and NP regions in 3-month-old HDAC9^KO^ mice was considered as the initiation of the repair process compared to HDAC9^WT^ mice and 1-month-old HDAC9^KO^ mice. However, proteoglycan content was notably decreased in 6-month-old HDAC9^KO^ mice (Fig. 3C). The histopathological scores based on the safranin-O/fast green staining showed that knockout of HDAC9 increased the IVD degeneration of lumbar in mice at 3 and 6 months of age (Fig. 3D). Statistically, the area ratios of NP cell band/IVD and NP/IVD were gradually decreased with aging in HDAC9^KO^ mice (Fig. 3E and F). During aging, there was no significant difference in the body weight between HDAC9^KO^ and HDAC9^WT^ mice at 1, 3, and 6 months of age (Fig. 3G). Having observed severe degeneration of IVDs in 6‐month‐old HDAC9^KO^ mice, we subsequently focused on the NP compartment. We confirmed that no expression of HDAC9 was observed in NP cells and there was an increase in TUNEL-positive cells in NP in HDAC9^KO^ mice compared with HDAC9^WT^ mice (Fig. 3H, I). These results indicate that IVDD is an age-related process and HDAC9 deletion accelerates the degenerative process in mice.

### MRNA-seq and Co-IP/MS analysis determine HDAC9-regulated genes and HDAC9-interacting proteins in mouse NP cells

**Fig. 4:**
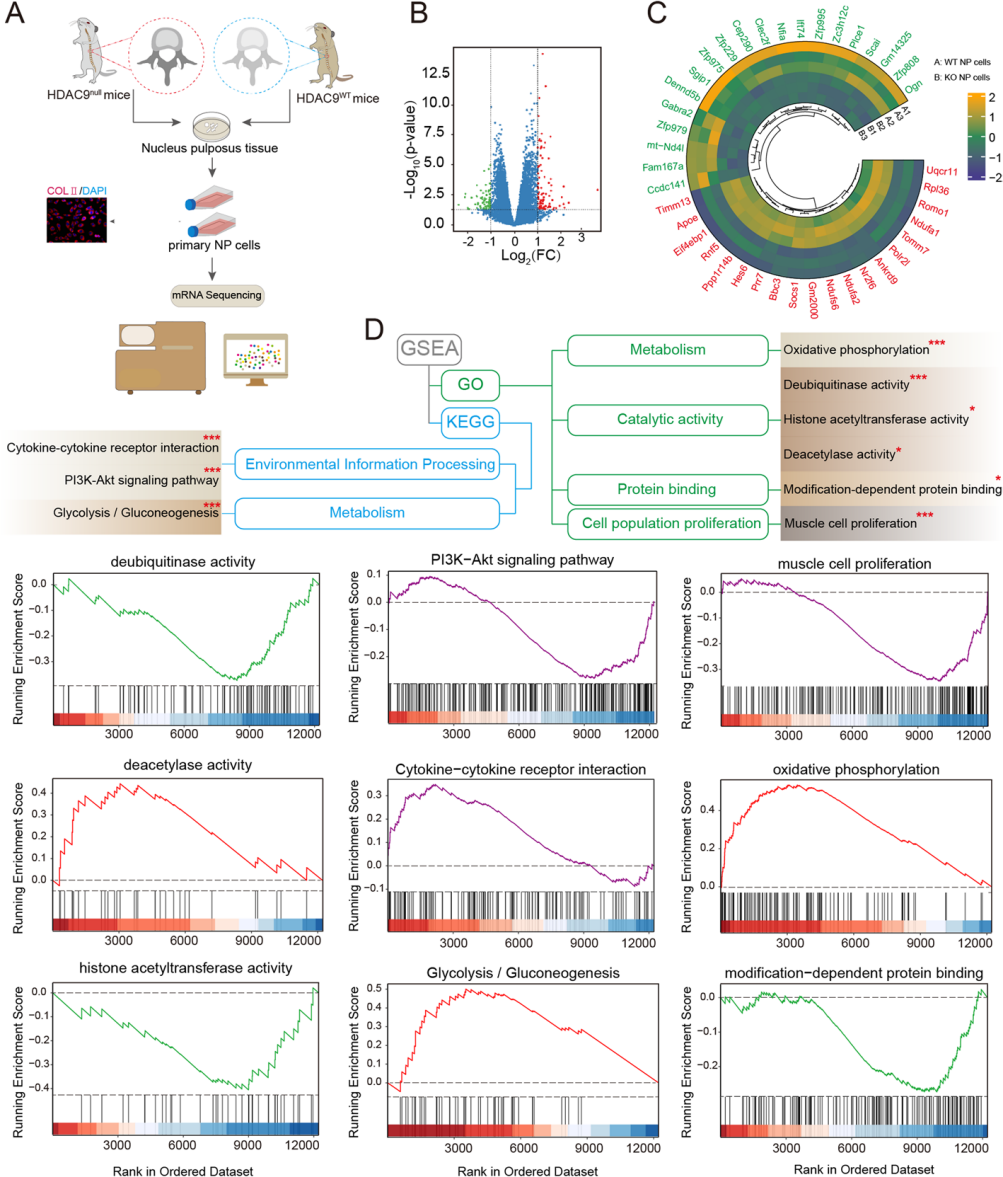
mRNA-seq analysis determines HDAC9-regulated genes in NP cells. **A** Schematic of the NP cells isolated from HDAC9^KO^ and HDAC9^WT^ mice (*n* = 3) were subject to mRNA-seq. **B** Volcano plot of expression of genes. Green, downregulated DEGs; red, upregulated DEGs; blue, not significant. **C** Heatmap of the expression profiling of the top 20 upregulated and downregulated DEGs. The red color and green color represent upregulated DEGs and downregulated DEGs, respectively. **D** Schematic of GSEA enrichment analysis of genes from mRNA-seq. GSEA plot depicting the enrichment of genes in deubiquitinase activity, PI3K-Akt signaling pathway, muscle cell proliferation, deacetylase activity, cytokine–cytokine receptor interaction, oxidative phosphorylation, histone acetyltransferase activity, glycolysis/gluconeogenesis pathway, and modification-dependent protein binding

To study the potential regulatory mechanism of HDAC9, the NP cells were isolated from HDAC9^WT^ and HDAC9^KO^ mice and identified by collagen II, and the RNA-seq analysis was performed (Fig. 4A). A total of 159 DEGs were identified (Fig. 4B). Of these, 89 genes were upregulated and 70 genes were downregulated in the HDAC9^KO^ NP cells compared with the control cells (Additional file 2: Table S2). The heatmap exhibited the top 20 upregulated and downregulated DEGs (Fig. 4C). To better understand the potential functions of genes in NP cells, GSEA enrichment analysis based on GO and KEGG was performed. GO analysis by GSEA indicated that the genes were mainly enriched in metabolism (oxidative phosphorylation), catalytic activity (deubiquitinase activity, histone acetyltransferase activity, deacetylase activity), protein binding (modification-dependent protein binding), and cell population proliferation (muscle cell proliferation). GSEA of KEGG pathways suggested that HDAC9 was related to cytokine–cytokine receptor interaction, PI3K-Akt signaling, and glycolysis/gluconeogenesis pathway (Fig. 4D).

**Fig. 5:**
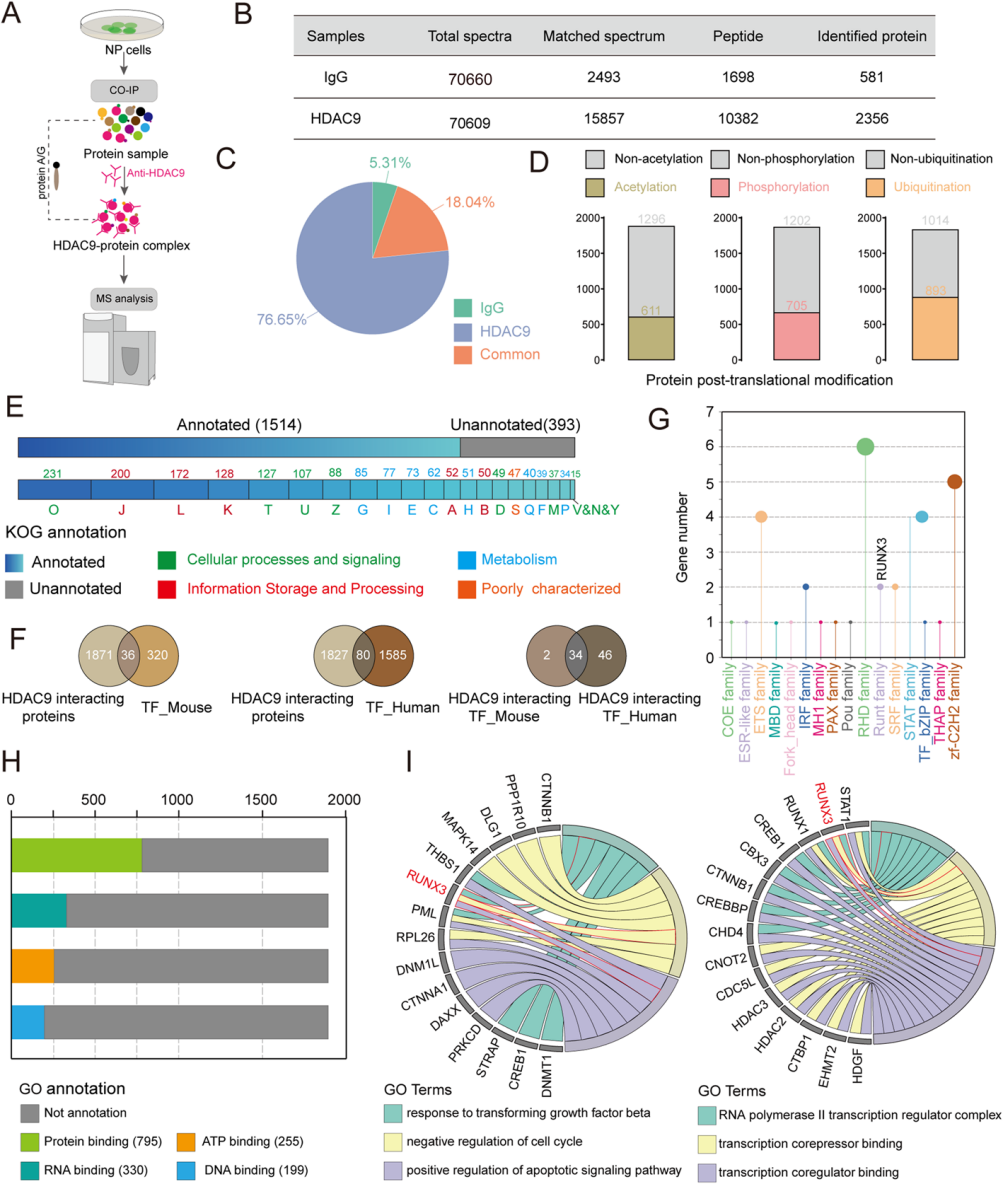
Co-IP/MS analysis determines HDAC9-interacting proteins in NP cells. **A** The NP cells isolated from C57BL/6J mice were subjected to co-IP/MS using anti-HDAC9 and IgG. **B**, **C** The protein identification results from database screening and the percentage of specific HDAC9-interacting proteins in NP cells. **D** The number of HDAC9-interacting proteins possibly modified by acetylation (611), phosphorylation (705), and ubiquitination (893) shown according to the PhosphoSitePlus database. **E** Eukaryotic Ortholog Groups (KOG) of proteins annotation of HDAC9-interacting proteins. **F** The number of HDAC9-interacting proteins that function as transcription factors in mice (36) and humans (80). **G** Lollipop plot presented the 34 common transcription factors in the classified 16 transcription factor families (Lollipop plot was drawn using https://www.chiplot.online/). **H**, **I** GO annotations determined the molecular functions of the HDAC9-interacting proteins

Next, we isolated NP cells from wild-type C57BL/6J mice and performed co-IP/MS analysis (Fig. 5A). A total of 1907 (76.65% of total protein) specific HDAC9-interacting proteins were identified (Fig. 5B, C, Additional file 3: Table S3). We searched all these proteins in the PhosphoSitePlus website and found that 611 of them contain acetylation sites, 705 of them contain phosphorylation sites, and 893 of them contain ubiquitination sites (Fig. 5D). Next, KOG annotation showed that 1514 HDAC9-interacting proteins were functionally and mainly classified into cellular processes and signaling, information storage and processing, and metabolism (Fig. 5E). We also analyzed the HDAC9-interacting proteins that function as transcription factor (TF) based on the human TF database (http://bioinfo.life.hust.edu.cn/HumanTFDB#!/) and mouse TF database (https://hocomoco11.autosome.org/). The 36 mouse TFs and 80 human TFs were included in HDAC9-interacting proteins, and 34 common TFs from mouse and human TFs were classified into 16 TF families (Fig. 5F, G). Moreover, GO annotation was performed on HDAC9-interacting proteins. We found that most of proteins participate in the binding function including protein binding, ATP binding, RNA binding, and DNA binding (Fig. 5H). GO analysis showed that HDAC9-interacting protein transcription factor RUNX3 from the Runt family in Fig. 5G is enriched in regulating negative regulation of the cell cycle, positive regulation of apoptotic signaling pathway, and transcriptional regulation (Fig. 5I). Interestingly, mRNA-seq analysis verified the mRNA expression of RUNX3 was not significantly changed between HDAC9^WT^ and HDAC9^KO^ NP cells. It suggested that HDAC9 may participate in cell apoptosis and vitality in NP cells via regulating RUNX3 expression through protein modification.

### Deficiency of HDAC9 induces cell apoptosis, inhibits cell viability in NP, and increases RUNX3 acetylation and expression

**Fig. 6:**
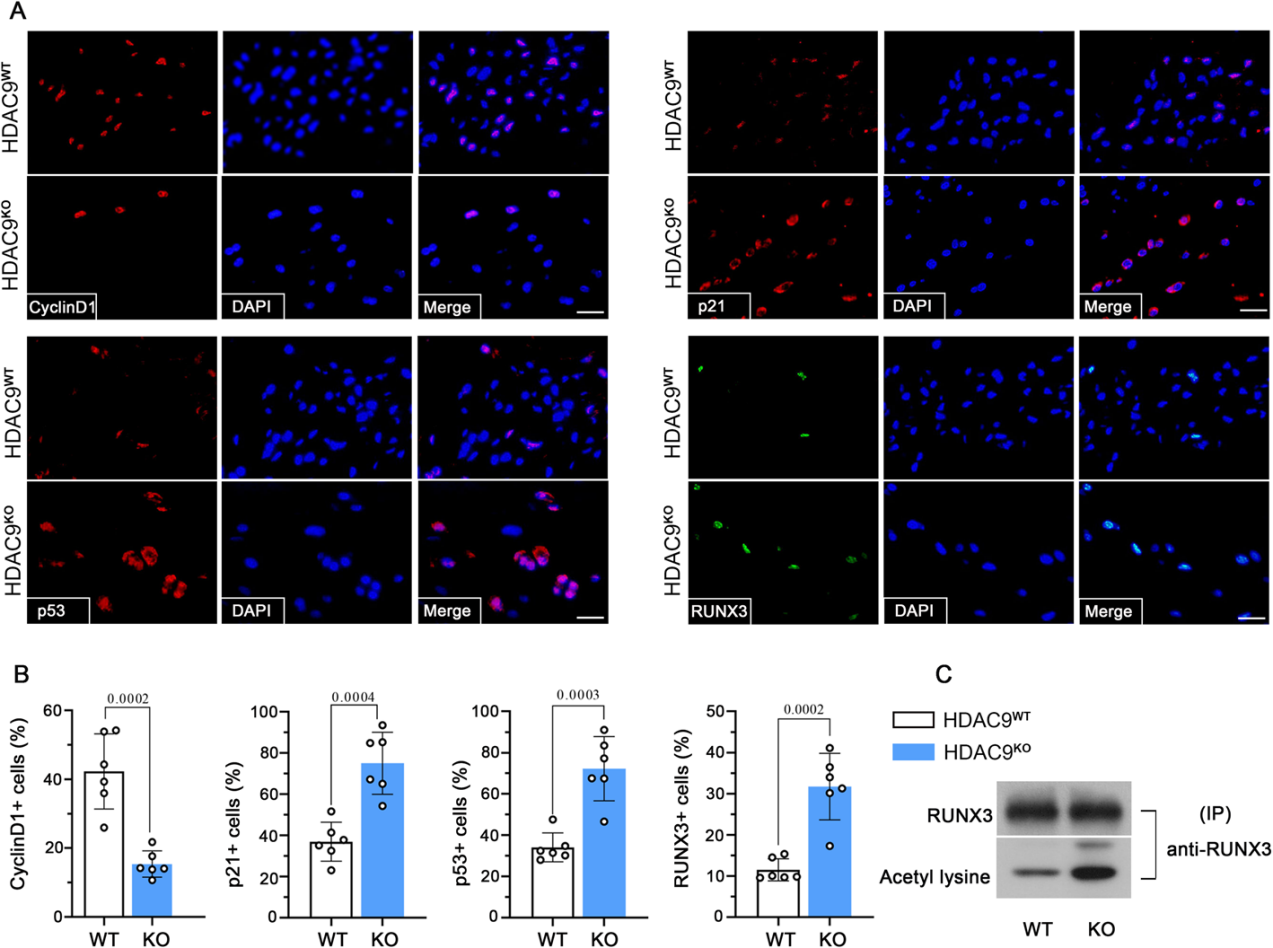
Deficiency of HDAC9 affects cell apoptosis, viability, and regulates RUNX3 in NP tissue. **A**, **B** Fluorescence staining and quantification of percentage of Cyclin D1 positive cells, p21 positive cells, p53 positive cells, and RUNX3 positive cells in the NP tissues of 6-month-old HDAC9^WT^ mice and HDAC9^KO^ mice. Scale bar, 25 μm. **C** The disc tissues were immunoprecipitated with anti-RUNX3 and acetylation of RUNX3 was analyzed using an anti-acetyl-lysine. Data are represented as mean ± SD (*n* = 6). A *p* value of less than 0.05 was considered significant using an unpaired Student’s *t*-test. NS, not significant

To further study the effect of HDAC9 on cell apoptosis and the vitality of NP cells, the expressions of cell cycle control factors and apoptotic factors were determined in the NP compartment. Knockout of HDAC9 reduced the expression of cell cycle-related protein cyclin D1 and significantly increased the cyclin-dependent kinase inhibitor p21. Moreover, HDAC9 deletion increased the expression of proapoptotic factor p53 and RUNX3 in NP cells (Fig. 6A, B). To further elucidate the mechanism underlying the regulation of HDAC9–RUNX3 on cell fate, the levels of acetylated RUNX3 were measured. The results showed that HDAC9 deletion observably increased the RUNX3 acetylation in IVD tissues (Fig. 6C). These results suggest that HDAC9 deletion may aggravate the degeneration of IVDs through decreasing cell viability and increasing cell apoptosis of the NP region. Additionally, HDAC9 may play its role in regulating the RUNX3 acetylation and expression.

### HDAC9 knockdown inhibits cell viability and induces the apoptosis of NP cells in vitro

**Fig. 7:**
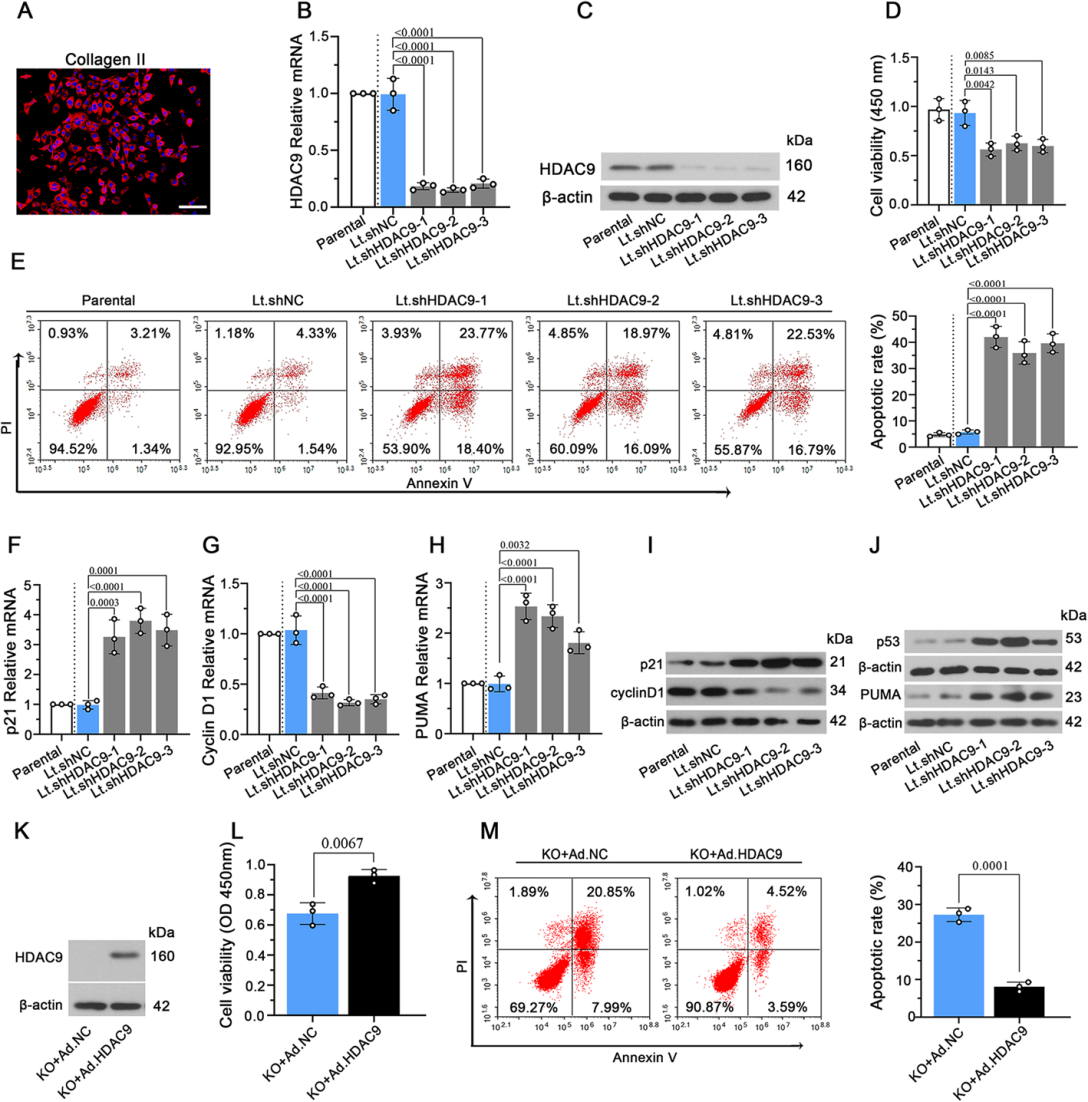
HDAC9 knockdown increases cell apoptosis and inhibits cell viability of NP cells in vitro. **A** Identification of isolated NP cells by collagen II using fluorescence staining. Scale bar, 100 μm. **B**, **C** NP cells were infected with lentivirus-mediated shHDAC9 (Lt.shHDAC9-1, 2, and 3) for 72 h. The relative mRNA level and protein level of HDAC9 in Lt.shHDAC9-infected NP cells were detected by RT–qPCR and western blot. **D** Cell viability of NP cells was measured by CCK-8 assay. **E** Apoptotic cells were stained with annexin V/propidium iodide and quantified by flow cytometry. The relative mRNA level of p21 (**F**), cyclin D1 (**G**), and PUMA (**H**). **I**, **J** The protein level of p21, cyclin D1, p53, and PUMA. **K** HDAC9^KO^ NP cells were infected with adenovirus containing cDNA of HDAC9 (Ad.HDAC9) for 72 h and the protein level of HDAC9 was detected. **L** Cell viability of NP cells was measured by CCK-8 assay. **M** Apoptotic cells were stained with annexin V/propidium iodide and quantified by flow cytometry. Data are represented as mean ± SD (*n* = 3). A *p*-value of less than 0.05 was considered significant using one-way ANOVA and Tukey’s multiple comparison test

To verify the effects of HDAC9 on NP cell viability and apoptosis, NP cells from C57BL/6J mice were identified by collagen II (Fig. 7A), and were infected with Lt.shHDAC9 and Lt.shNC for 72 h to downregulate HDAC9 expression. HDAC9 was knocked down successfully in NP cells by Lt.shHDAC9, which was confirmed by RT–qPCR and western blot (Fig. 7B, C). HDAC9 knockdown significantly inhibited cell viability and promoted cell apoptosis (Fig. 7D, E). RT–qPCR showed increased mRNA levels of *p21* and *PUMA* and reduced levels of *cyclinD1* in Lt.shHDAC9 cells compared to Lt.shNC cells (Fig. 7F–H). Moreover, the protein level of cyclin D1 was decreased and p21 was increased after HDAC9 knockdown, and protein levels of p53 and PUMA were enhanced in HDAC9 knockdown cells (Fig. 7I, J). Our results indicate that HDAC9 knockdown inhibits viability and induces the apoptosis of NP cells in vitro.

Next, HDAC9 was overexpressed in HDAC9^KO^ NP cells using adenovirus, and the effect of HDAC9 overexpression on cell viability and apoptosis in HDAC9^KO^ NP cells was determined. The protein expression of HDAC9 in HDAC9^KO^ NP cells after 72 h infection was detected in Fig. 7K. Overexpression of HDAC9 increased the cell viability and reduced the rate of apoptotic cells in Ad.HDAC9-infected cells compared with Ad.NC-infected cells (Fig. 7L, M). The results indicate that overexpression of HDAC9 could inhibit the apoptosis and loss of cell viability in HDAC9^KO^ NP cells.

### HDAC9 regulates cell viability and induces apoptosis in NP cells via mediating acetylation and ubiquitin–proteasomal degradation of RUNX3

**Fig. 8:**
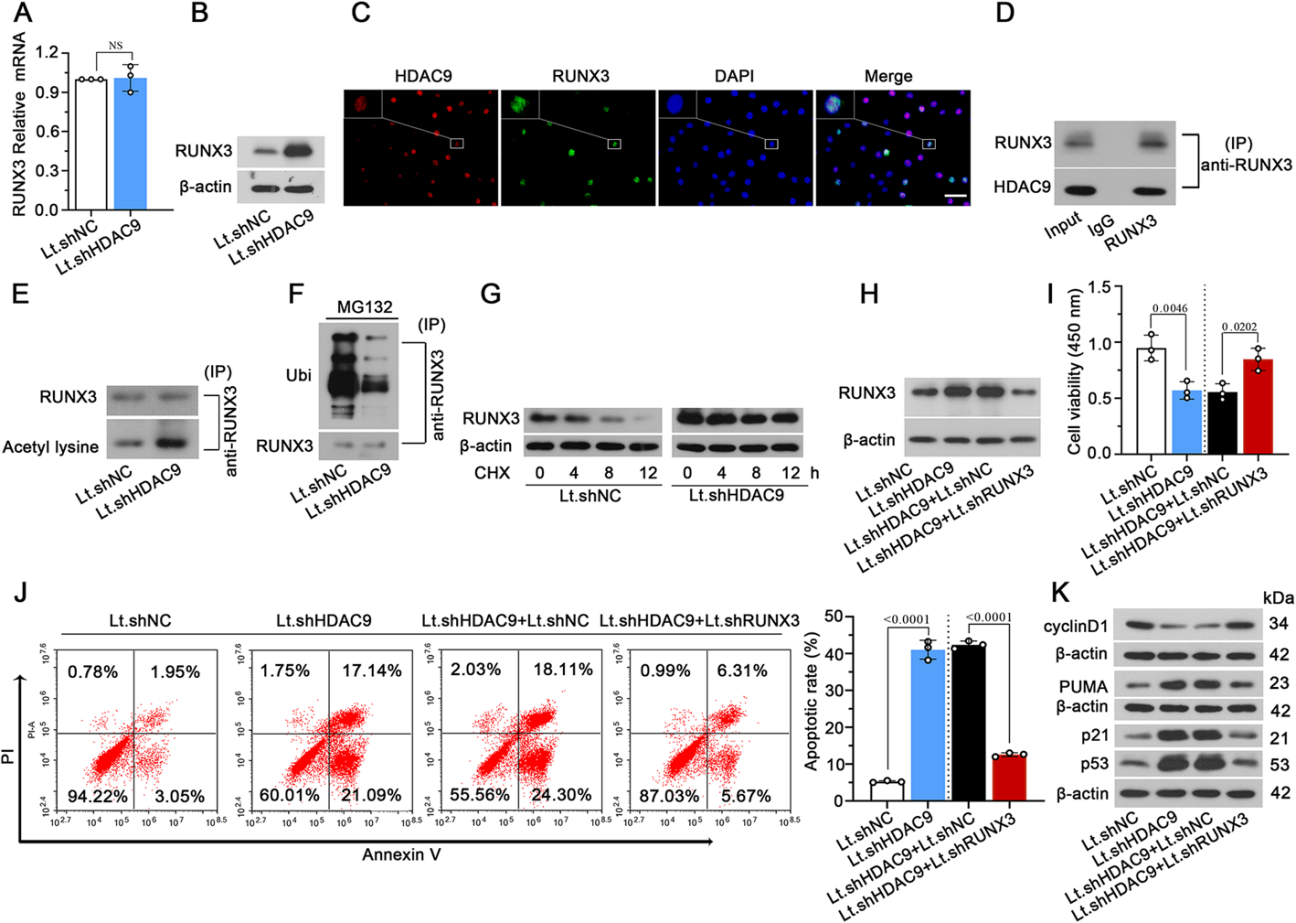
Knockdown of HDAC9 increases acetylation and inhibits ubiquitin–proteasomal degradation of RUNX3. **A**, **B** The mRNA and protein level of RUNX3 in Lt.shHDAC9-infected NP cells. **C** Double fluorescence staining of HDAC9 and RUNX3 in isolated NP cells. Scale bar, 50 μm. **D** NP cells were immunoprecipitated with anti-RUNX3 to analyze of the interaction of HDAC9 and RUNX3. **E** The acetylation of RUNX3 in HDAC9 knockdown cells. **F** Lt.shHDAC9-infected cells were treated with MG132 for 8 h and immunoprecipitated with anti-RUNX3 to detect ubiquitination of RUNX3 using anti-ubiquitin (ubi) antibody. **G** Lt.shHDAC9-infected cells were treated with cycloheximide (CHX) for indicated time points, and then RUNX3 remaining protein level was detected by western blot. **H** NP cells were infected with Lt.shHDAC9 and Lt.shRUNX3 for 72 h and the protein level of RUNX3 was detected. **I** Cell viability of NP cells was measured by CCK-8 assay. **J** Apoptotic cells were stained with annexin V/propidium iodide and quantified by flow cytometry. **K** The protein level of p53, p21, PUMA and Cyclin D1 were detected by western blot. Data are represented as mean ± SD (*n* = 3). A *p* value of less than 0.05 was considered significant using one-way ANOVA and Tukey’s multiple comparison test

To determine the molecular mechanism, we studied the effect of HDAC9 on RUNX3 acetylation and expression in NP cells. The mRNA level of RUNX3 did not change, but the protein level was decreased in HDAC9 knockdown cells (Fig. 8A, B). Moreover, HDAC9 and RUNX3 were coexpressed and physically interacted in NP cells (Fig. 8C, D). Knockdown of HDAC9 significantly increased RUNX3 acetylation in NP cells (Fig. 8E). To further assess whether HDAC9 affects the expression of RUNX3 via ubiquitin–proteasomal degradation, a proteasome inhibitor MG132 was used to block the proteasomal degradation in Lt.shHDAC9 cells. The result showed that RUNX3-conjugated ubiquitin was decreased in Lt.shHDAC9 cells under MG132 treatment (Fig. 8F). Moreover, protein degradation of RUNX3 was remarkably reduced in Lt.shHDAC9 cells compared with Lt.shNC cells under protein synthesis inhibitor cycloheximide (CHX) treatment (Fig. 8G). The downregulation of RUNX3 by Lt.shRUNX3 could prevent Lt.shHDAC9-induced RUNX3 upregulation (Fig. 8H). RUNX3 silence reversed the effect of HDAC9 knockdown on cell viability and apoptosis and protein expression of p21, cyclin D1, p53, and PUMA (Fig. 8I–K). The results suggest that the knockdown of HDAC9 reduced cell viability and induced apoptosis in NP cells by enhancing RUNX3 expression.

### Overexpression of HDAC9 alleviates surgery-induced IVDD in vivo

**Fig. 9:**
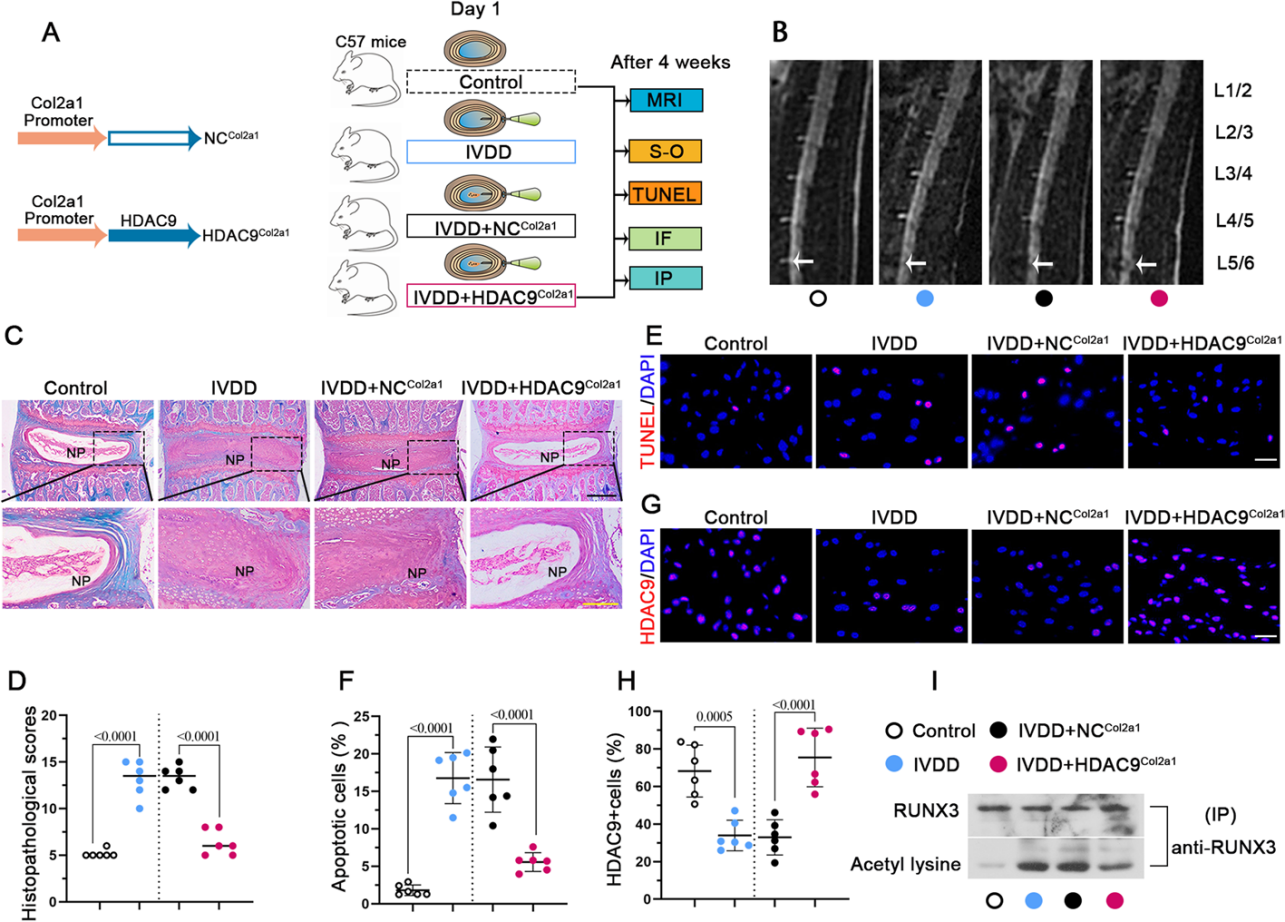
Overexpression of HDAC9 alleviates surgery-induced IVDD in mice. **A** Schematic of HDAC9 overexpression and animal working model. Surgically induced IVDD model was induced by puncturing at the L5/6 IVD with a 30-gauge needle, and adenovirus carrying Col2a1-promoter mediated overexpression of HDAC9 (HDAC9^Col2a1^) and NC^Col2a1^ were injected to the NP tissue during needle puncture. After 4 weeks of surgery, the mice were sacrificed. **B** MRI scan of the lumbar spine. **C**, **D** Coronal sections of disc compartments stained and analyzed by safranin-O/fast green. Scale bar: black, 500 μm; yellow, 200 μm. **E**, **F** Apoptotic cells in the NP compartment were detected by TUNEL staining. Scale bar, 25 μm. **G**, **H** Fluorescence staining of HDAC9 in NP compartment. Scale bar, 25 μm. **I** The acetylation level of RUNX3 in disc tissues. Data are represented as mean ± SD (*n* = 6). A *p* value of less than 0.05 was considered significant using one-way ANOVA and Tukey’s multiple comparison test

Finally, the potential therapeutic effect of HDAC9 overexpression on surgery-induced IVDD progression in vivo was determined. HDAC9 was specifically overexpressed by adenovirus carrying Col2a1 promoter and CDS of HDAC9 (HDAC9^Col2a1^) and injected into NP tissue during the needle puncture. After 4 weeks, we assessed the effects of HDAC9 on surgery-induced IVDD (Fig. 9A). We reviewed the MRI results of IVD with disc puncture surgery. The MRI image revealed that the intensity of L5/6 after puncture was higher in the HDAC9-overexpressed HDAC9^Col2a1^ mice than in the NC^Col2a1^ mice (Fig. 9B). The safranin-O/fast green and TUNEL staining confirmed that the extracellular matrix (ECM) degradation and apoptosis of IVD were reduced after HDAC9 overexpression (Fig. 9C–F). Immunofluorescence staining showed that the expression of HDAC9 in the NP tissues was higher in the IVDD + HDAC9^Col2a1^ group than in the IVDD + NC^Col2a1^ group (Fig. 9G, H). Additionally, acetylation of RUNX3 was decreased in IVDD + HDAC9^Col2a1^ group (Fig. 9I). Collectively, these results suggested that overexpression of HDAC9 in NP cells alleviated surgery-induced IVDD in mice model.

## Discussion

IVDD is a chronic process that involves many triggering events including mechanical trauma, genetic disposition, infection, and age [33]. Aging is an important impactor on IVDD. Kadow et al. have summarized that the number of vascular channels in the EP region decreases with aging, which limits the diffusion of nutrients into IVDs and the removal of waste products. Nutrient deficiency and oxidization of protein and lipid will cause inflammation, oxidative stress, mitochondrial dysfunction, and DNA damage, thus driving cell death in the discs, especially in AF and NP tissues [34–36]. Recently, gene-based therapy and studies have proved several genes play crucial roles in the regulation of the apoptosis of NP cells in IVDD [37]. For instance, Alvarez‐Garcia et al. found that conditional deletion of forkhead box protein (FOXO) isoforms (FOXO1, 3, and 4) causes IVDD in aging mice and FOXO1/3 is required to protect NP cells from apoptosis [38]. Additionally, a specific circular RNA CircGLCE is downregulated in NP tissues of IVDD patients and overexpression of CircGLCE alleviates IVDD in vivo by suppressing the apoptosis of NP cells and degradation of ECM [39]. Bioinformatic analysis in this study suggests that protein modification is important for the procession of IVDD. HDAC9 is decreased in NP tissues in patients with IVDD, and HDAC9 functions as histone deacetylase and correlates with lots of DEGs in NP tissues. We also found that HDAC9 expression was decreased in degenerated IVD in 18-month-old mice and HDAC9 deletion aggravated the degeneration of IVD and promoted the apoptosis of NP cells. In isolated NP cells, we confirmed that downregulation of HDAC9 reduced cell viability and induced the apoptosis, and overexpression of HDAC9 partially rescued the reduced cell viability and increased apoptosis in HDAC9^KO^ NP cells. Overexpression of HDAC9 in collagen II-expressed NP cells alleviated the IVDD in mice. Moreover, we found that the HDAC9-interacting proteins could be enriched in the apoptotic signaling pathway or cell cycle pathway. The results indicate that HDAC9 is required to protect cell survival and inhibit apoptosis of NP cells.

HDAC9 is considered an oncogene in many kinds of cancer including oral squamous cell carcinoma, gastric cancer, lung cancer, and pancreatic ductal adenocarcinoma [27–29, 40]. Additionally, the knockdown of HDAC9 increases the apoptosis in the serum-deprived GT1-7 GnRH neuronal cell line [25]. Previous studies suggest that regulation of protein acetylation status by HDACs could promote or inhibit protein ubiquitination and the subsequent ubiquitin–proteasomal degradation [41–43]. The roles of HDAC9 in regulating gene expression and function have been reported. As one of the histone deacetylases, HDAC9 is involved in histone deacetylation at the promoter of target genes and then suppresses gene transcription. For example, HDAC9 deficiency increased the expression of ATP binding cassette subfamily A member 1 (ABCA1) and peroxisome proliferator-activated receptor gamma (PPARγ) by accumulating acetylated histones at the promoters of ABCA1 and PPARγ in mouse macrophages [44]. However, HDAC9 may also help deacetylation of non-histone proteins such as transcription factors to regulate their activity and downstream target gene expression. Upstream stimulatory factor 1 (USF1), for instance, is a non-histone substrate of HDAC9 and overexpression of HDAC9 reduces the acetylation and transcriptional activity of USF1 in HEK293F cells [45]. HDAC9 also could interact with other proteins like ataxia-telangiectasia group D complementing (ATDC) to inhibit its acetylation but not expression [22]. Choi et al. found that HDAC9 reduced the acetylation of Nkx3.2, leading to its sumoylation and proteasomal degradation [46]. Previous studies suggested that the same HDAC-mediated acetylation could promote or inhibit the ubiquitination and degradation of the target protein. Knockdown of HDAC6 increased the acetylation of heat-shock protein 5 (HSPA5) and reduced the ubiquitination and degradation of HSPA5 in breast cancer cells [43]. However, HDAC6 also can deacetylate p62, and acetylation of p62 promotes its ubiquitin–proteasomal degradation [42]. It indicates that the effect of HDACs on the expression of protein should be considered individually, and this might depend on the regulation between the acetylation and ubiquitination for each target protein.

In this study, our results showed that HDAC9 interacts with RUNX3 in NP cells and RUNX3 has the cell functions in negative regulation of cell cycle and positive regulation of apoptotic signaling pathway determined by co-IP/MS. HDAC9 deletion increased RUNX3 acetylation and expression in NP tissues in vivo. Moreover, the knockdown of HDAC9 increased RUNX3 acetylation and decreased ubiquitin–proteasomal degradation in NP cells in vitro. The RUNX3 is a transcription factor expressed in many tissues and various types of cells and exhibits a broad range of biological functions. RUNX3 acetylation inhibits its ubiquitination-mediated degradation and promotes its transactivation activity [47, 48]. In lung cancer cell lines, RUNX3 increases the expression of p21 and inhibits cell proliferation [49]. RUNX3 induces cell cycle arrest and inhibits cell proliferation of gastric cancer cells by inactivating Akt1/β-catenin/cyclin D1 signaling pathway [50]. In addition, acetylated RUNX3 can interact with p53, which induces the phosphorylation of p53 at Ser-15 and inhibits mdm2-mediated p53 degradation. Overexpression of RUNX3 promotes the endogenous expression of p53 downstream such as p21, PUMA, and BAX [51]. PUMA is an important proapoptotic molecule [52]. PUMA is overexpressed in lumber IVDs of aged rats and involved in apoptosis of human NP cell line HNPC cells [53]. We found that RUNX3 was upregulated in degradative IVD in HDAC9^KO^ mice and knockdown of RUNX3 increased the cell viability and reduced the apoptosis in Lt.shHDAC9-infected NP cells. These results indicate that the downregulation of HDAC9 may reduce cell viability and induce apoptosis in IVDs by enhancing RUNX3 expression. One of the limitations of this study is that, instead of identification of the differentially expressed proteins regulated by HDAC9 via quantitative proteomics, here we chose RUNX3 as the candidate based on co-IP/MS analysis. We will uncover more potential targets of HDAC9 in the nucleus pulposus by conducting quantitative proteomics in the future. Additionally, we only performed the gene therapy using an adenovirus-based vector to overexpress HDAC9 in the nucleus pulposus in a mouse model. There is still a long way for HDAC9 as a therapeutic target in clinical intervention for IVDD. As technology advances, many non-viral gene therapy approaches, such as liposomes [54], mesenchymal stem cell-derived exosomes [55], and microsphere-based delivery system [56], may be clinically applied instead of virus vector. More potential therapeutic targets such as HDAC9 will possibly be used in the clinical treatment of IVDD.

## Conclusions

In summary, our results suggest that HDAC9 plays an important role in the development and progression of IVDD. It might be required to protect NP cells against the loss of cell viability and apoptosis by inhibiting RUNX3 acetylation and expression during IVD degeneration. Our findings may help provide a better understanding of the course of IVDD and offer a potential therapeutic direction.

### Supplementary Information

**Additional file 1. **Correlation analysis of the six deacetylation genes, CHD3, HDAC9, LYPLA1, HDAC3, LYPLAL1 and SIRT5 with the DEGs from the GSE56081 dataset.

**Additional file 2. **The differentially expressed genes (DEGs) between HDAC9^KO^ NP cells and HDAC9^WT^ NP cells from mRNA-seq analysis.

**Additional file 3. **The HDAC9-interacting proteins in NP cells from co-IP/MS analysis.

## Data Availability

The datasets used and/or analyzed during the current study are available from the corresponding author on reasonable request.

## Abbreviations

Ad: Adenovirus
AF: Annulus fibrosus
BSA: Bovine serum albumin
BP: Biological process
CC: Cellular component
CHX: Cycloheximide
Co-IP: Co-immunoprecipitation
EP: Endplate
FBS: Fetal bovine serum
GEO: Gene Expression Omnibus
GO: Gene Ontology
HAT: Histone acetyltransferase
HDACs: Histone deacetylases
HDAC9: Histone deacetylase 9
IVD: Intervertebral disc
IVDD: Intervertebral disc degeneration
KEGG: Kyoto Encyclopedia of Genes and Genomes
KO: Knockout
LBP: Low-back pain
MF: Molecular function
MRI: Magnetic resonance imaging
MS: Mass spectrometry
NC: Negative control
NP: Nucleus pulposus
PI: Propidium iodide
RT–qPCR: Quantitative real-time reverse transcription polymerase chain reaction
RUNX3: RUNX family transcription factor 3
sgRNA: Small guide RNA
TF: Transcription factor
TUNEL: Terminal deoxynucleotidyl transferase dUTP nick-end labeling
WT: Wild type
